# Disease Refuge or Ecological Trap: Location‐Specific Performance of Amphibian Hotspot Shelters

**DOI:** 10.1002/ece3.72590

**Published:** 2026-03-30

**Authors:** Madeleine L. Holmes, Shannon W. Kaiser, Sneha S. Basu, Lee Berger, Lee F. Skerratt, Richard Shine, Anthony W. Waddle

**Affiliations:** ^1^ School of Natural Sciences Macquarie University Sydney New South Wales Australia; ^2^ Melbourne Veterinary School University of Melbourne Melbourne Victoria Australia; ^3^ Applied Biosciences Macquarie University Sydney New South Wales Australia

**Keywords:** *Batrachochytrium dendrobatidis*, behavioural thermoregulation, chytridiomycosis, ecological trap, habitat manipulation, pathogens, thermal mosaic, thermal refugia

## Abstract

Many terrestrial ectotherms (amphibians, reptiles) select warm retreat sites which may provide opportunities to escape the impacts of pathogens that cannot tolerate high temperatures. Recent research has exploited this mismatch in host‐pathogen thermal tolerance by providing sun‐heated artificial hotspot shelters to protect endangered frogs from the amphibian chytrid fungus (*Batrachochytrium dendrobatridis*), which causes the disease chytridiomycosis. Artificial shelters comprised of masonry bricks and small greenhouses allow endangered frogs to stay warm in winter, reducing and clearing chytrid infections and developing resistance to future infection even under cold temperatures. Such shelters can be a valuable tool for conservation in sunny cool weather, but cloudy weather reduces the thermal benefit; and in too‐cool conditions the warming creates a thermal regime where chytrid could grow faster. We monitored temperatures inside hotspot shelters constructed at two sites in Australia over winter: one in a cool semi‐arid climate (Werribee, Victoria, Australia), and one in a humid subtropical climate (Sydney, NSW, Australia), to evaluate the thermal performance of shelters in different chytrid‐impacted climates. Our monitoring of temperatures inside hotspot shelters found minor‐to‐negative outcomes at the cooler site, with shelters in Werribee exceeding 25°C for at least 1 h on only 13.1% of days, compared to 82.5% of days at the Sydney site. Hotspot shelters have great potential for frog conservation, but they are not a panacea because diversity in habitat conditions and target species responses will influence their success.

## Graphical abstract

Artificial hotspot shelters, which provide sun‐heated retreats, can help frogs clear infections from the amphibian chytrid fungus (*Batrachochytrium dendrobatidis*) by creating warm microhabitats unsuitable for the pathogen. We monitored shelter temperatures at two Australian sites and found they reached therapeutic levels far more often in Sydney than in cooler Werribee, suggesting local climate strongly influences their thermal performance and conservation potential.

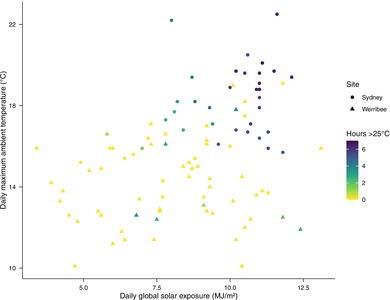

## 1 Introduction

Population declines of amphibians worldwide have stimulated research to mitigate threatening processes (Clulow et al. 2018; Kosch et al. 2022; Scheele et al. 2021). One such threatening process is the amphibian disease chytridiomycosis, caused by the fungal skin pathogen *Batrachochytrium dendrobatidis* (*Bd*) (Scheele et al. 2021). *Bd* has been introduced globally, likely through the pet trade (Fu and Waldman 2022). *Bd* is associated with seasonal mass die‐off events (Berger et al. 2004), population declines, and seven species extinctions in Australia (Scheele et al. 2017). Species that inhabit cool and high‐elevation areas are overrepresented in terms of losses attributed to *Bd* globally (Scheele et al. 2019), suggesting a relationship between environmental conditions and disease susceptibility in *Bd*‐impacted amphibians.

Two central features of terrestrial ectotherms are (1) their dependence upon ambient thermal heterogeneity to select body temperatures that enhance fitness (Pough 1980) and (2) the low energy costs of inactivity, with individuals spending long periods inactive within secure retreat sites (Pough 1980). The thermal conditions within a retreat site may affect fitness‐relevant traits such as the ability to detect predators, to digest prey, to mount an immune response and to suppress heat‐intolerant pathogens (George et al. 2017; Puschendorf et al. 2011; Rollins‐Smith 2020; Stevenson et al. 2013). Ironically, although warmer climates may imperil many ectotherms via overheating (Kearney et al. 2009), other taxa may be threatened by a lack of access to heat. For example, forest thickening and weed invasion can eliminate thermally suitable refuges (Croak et al. 2013).

In response to anthropogenic loss of warm retreat‐sites, reptile researchers have enhanced thermal regimes by removing vegetation (Morrison 2013) and/or by deploying artificial retreat‐sites (Croak et al. 2013). Such manipulations have rarely been attempted for amphibians because these animals tend to select low rather than high body temperatures (Tracy et al. 2010). However, some frogs bask by day, and prefer warm conditions (Hamer et al. 2003). Indeed, natural areas with less canopy cover and greater basking opportunities appear to buffer frog populations against infectious disease (Puschendorf et al. 2011). Thus, warm refuges might benefit threatened amphibians as well as reptiles (Puschendorf et al. 2011; Scheele et al. 2019).

A recent study demonstrated remarkable success in this endeavour. Waddle et al. (2024) created hotspot shelters in Sydney by constructing brick piles within small greenhouses inside outdoor mesocosms (miniature habitat). The mesocosms were comprised of 10,000 L aquaculture tubs that were partially filled with gravel and water to mimic a pond's edge. Green and Golden Bell Frogs ( *Litoria aurea* ) were added to each mesocosm, half of which were experimentally infected with *Bd*. All mesocosms contained brick piles inside greenhouses, but half of the mesocosms had their greenhouses covered with 90% shade cloth to reduce passive warming capacity. Frog body temperatures were an average of 3.1°C higher in the mesocosms with unshaded hotspots, and *Bd* infection loads in these mesocosms were substantially lower (Waddle et al. 2024).

Broadly, *Bd* grows at an increasingly rapid rate over the thermal range from 10°C to 25°C, with growth decreasing between 25°C and 30°C, reaching its critical thermal maximum at approximately 30°C in vitro (Stevenson et al. 2013; Voyles et al. 2017). Reported in vivo thermal performance of the pathogen is varied and can differ from in vitro performance; Cohen et al. (2017) simultaneously grew two *Bd* isolates both in culture and on live frogs, finding that growth in culture was closely matched to previously reported in vitro thermal performance, with growth rates peaking between 18°C and 19°C. However, when tested in vivo, peak growth occurred at either 10°C or 14°C on *Anaxyrus terrestris* and *Osteopilus septentrionalis*, respectively. Additionally, peak *Bd* growth on *Atelopus zeteki* occurred at 26°C, despite the same isolate showing peak growth at 18°C in vitro. *A. terrestris* and *O. septentrionalis* are both adapted to warm, lowland subtropical habitats, whilst *A. zeteki* is adapted to cool high‐elevation cloud forests. This reported discrepancy between in vitro and in vivo thermal performance likely reflects reduced immune function in the frogs when kept at temperatures to which they are not physiologically adapted (Rollins‐Smith 2020), rather than changes in thermal performance of the pathogen itself. These reported discrepancies may also reflect interactions between *Bd* and other skin microbiota in vivo that are not typically included in in vitro testing. Indeed, the skin microbiome has been shown to be protective against chytridiomycosis in some amphibian species (Rebollar et al. 2020; Woodhams et al. 2023), and this interaction is likely to be influenced by temperature as well.

Whilst in vitro growth rates do not necessarily directly translate to in vivo growth rates, it is nevertheless likely that hotspot shelters will be ineffective in cool climates where low ambient temperatures may already be slowing *Bd* growth (i.e., remaining below 10°C, see: Piotrowski et al. 2004; Stevenson et al. 2013; Cohen et al. 2017), and local frogs are adapted to such cool temperatures. More worryingly, shelters in such a climate may elevate temperatures to a range that favours accelerated growth of the pathogen, thus forming an ‘ecological trap’—whereby a trait that enhances fitness under previous selective regimes (i.e., selection of the warmest available retreat site) imperils the animal in a habitat in which *Bd* occurs (Schlaepfer et al. 2002). This is especially the case for species that do not thermoregulate in response to infection, instead preferring warmer retreat sites when available regardless of infection status (Sauer et al. 2018; Waddle et al. 2024). However, cool climates may still have high levels of daily solar radiation exposure, and shelters designed to heat up passively via sunlight exposure may increase microhabitat temperatures substantially (Mussard 2017). To prevent the inadvertent creation of ecological traps, we need to determine how shelters, such as those deployed by Waddle et al. (2024), will perform across a variety of climatic conditions and with a diversity of target species. Hence in this study we analysed the temperatures in sun‐heated artificial hotspot shelters in mid‐winter, the period when ambient conditions are coolest and frogs have the least access to naturally warm retreats. This timing captures the season of greatest *Bd* risk and provides the most informative assessment of whether hotspot shelters can mitigate infection under limiting thermal conditions. We compared shelters at a sub‐tropical Sydney site (where shelters were previously shown to be effective in reducing *Bd* infections) and a higher latitude temperate site in Werribee, Melbourne, to assess potential influence on disease outcomes in a cooler climate (Figure S1).

## 2 Materials and Methods

### 2.1 Design of Hotspot Shelters

We constructed artificial refuge‐sites from house‐bricks (each 230 × 110 × 75 mm, containing 10 circular holes 30 mm in diameter stacked into piles of 10, in four levels: Figure S2). A greenhouse (Greenlife Square Drop Over Greenhouse with PE Cover, 1850 × 850 × 1020 mm) was erected over each shelter. We deployed two thermal dataloggers (Thermochron iButtons model DS1921G‐F5; Maxim Integrated Products, Sunnyvale, CA) in the holes within bricks in each shelter to monitor thermal regimes, one in the lowest layer of bricks and one in the second highest layer, in contact with the bricks. We also deployed dataloggers under rectangular wooden boards (660 × 95 mm, 30 mm thick) placed on the ground beside the greenhouses to measure temperatures in the kind of retreat‐site often used by amphibians (Hamer et al. 2003). Dataloggers were programmed to measure and record temperature every 30 min.

Three shelters containing two dataloggers each were set up on the Werribee campus of the University of Melbourne (37.8899° S, 144.6934° E, 35 m elevation) during June and July of 2022 across two monitoring periods (18 June 2022 to 6 July 2022, and 10 July to 20 August 2022—60 days). The region is characterised as a cold semi‐arid climate (Beck et al. 2018) and the site was selected for its minimal tree cover to maximise daily sun exposure and enable assessment of optimal thermal performance in a cool temperate climate. Three equivalent shelters, constructed in the same way, had been set up during previous years as part of the study by Waddle et al. (2024) within outdoor mesocosms (circular polyethylene tubs 3.6 m in diameter and 1000 mm high) in the Fauna Park at Macquarie University, Sydney (33.7968° S, 151.1244° E, 50 m elevation, humid subtropical) from 10 June to 19 July 2021 (40 days); data from these Sydney shelters were used for illustrative comparison with the Werribee site.

Monitoring was conducted mid‐winter to capture the period when ambient temperatures are typically lowest and thermal refugia are most limited. This timing aligns with the seasonal peak of chytridiomycosis risk in Australia (Berger et al. 2004), when sustained cool and moist conditions presumably favour *Bd* persistence. Although amphibian activity decreases in winter, several temperate species remain intermittently active or may shift microhabitats during brief warm periods. The shelters therefore represent potential refugia that frogs could realistically encounter or occupy during the winter period.

### 2.2 Data Analysis

We analysed data on thermal regimes using R version 4.3.1 (R Core Team 2025). To quantify operative temperatures, we calculated the number of hours per day above thresholds of 20°C, 25°C and 30°C. These thresholds are biologically relevant; for example, *Bd* declines in viability above 25°C and is rarely reported to survive above 30°C (Stevenson et al. 2013). We therefore used 25°C as a conservative benchmark for conditions likely to suppress *Bd*, while recognising that exposure duration and preceding cooler periods likely influence outcomes. Both infection prevalence and load decrease significantly in *L. aurea* populations at ambient temperatures above 25°C (Clulow et al. 2018; Waddle et al. 2024). More generally, many performance traits in ectotherms—including locomotion, metabolic rate, immune function and reproductive output—are substantially affected over this thermal range, typically improving with increasing temperature up to an optimal point (Angilletta 2006; Clusella‐Trullas and Chown 2014). We also calculated the daily average maximum and minimum temperatures across the structures at each site. We then compared these temperatures to the daily solar exposure (MJ/m^2^), rainfall amount (mm), and maximum and minimum temperature (°C) recorded at the closest Australian Bureau of Meteorology (BOM) weather station to each site that had available data for the period of interest. Weather station details are available in Tables S1 and S2. Corresponding data from each station can be accessed from the Australian Government Bureau of Meteorology Climate Data Online portal at http://www.bom.gov.au/climate/data/.

Because the Sydney and Werribee trials were conducted in different years and under distinct climatic conditions, we did not perform direct statistical comparisons between sites. Instead, we evaluated how daily shelter and amphibian retreat analogue (logger beneath a wooden board) temperatures responded to concurrent weather conditions within each site. We fitted generalised least‐squares (GLS) models with an autoregressive correlation structure (AR(1)) to account for temporal autocorrelation among days. Daily maximum shelter and wood temperatures were modelled as functions of ambient maximum temperature (°C) and daily solar exposure (MJ/m^2^). Equivalent models examined the number of hours per day that shelter temperatures exceeded 25°C. Analyses were conducted separately for each site, and model slopes were used to quantify how strongly microhabitat temperatures tracked variation in local weather.

## 3 Results

### 3.1 Sydney Site

**FIGURE 1:**
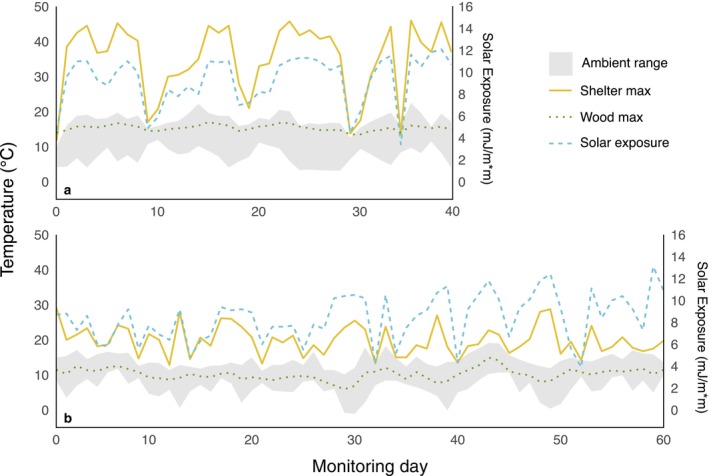
Solar exposure and temperatures from Sydney (a) and Werribee (b) across the winter monitoring periods. These times were 10 June to 19 July 2021 in Sydney, and 18 June 2022 to 6 July 2022 then 10 July to 20 August 2022 in Werribee. Temperature is on the left axis and solar exposure is on the right axis. The grey shaded area shows the range between minimum and maximum daily ambient temperature, yellow solid lines are maximum daily average shelter temperature, blue dashed lines are daily solar exposure, and green dotted lines are average maximum daily temperature beneath wood.

The average maximum daily ambient temperature recorded was 17.6°C (SD = 2.3°C), with the highest daily maximum reaching 22.5°C. The average daily minimum temperature was 7.1°C (SD = 2.6°C), with the lowest daily minimum being 3.2°C. Temperatures beneath wooden boards on the ground never reached 25°C, with the average maximum daily temperature reaching only 15.4°C (SD = 0.9°C). The highest average maximum daily temperature recorded within the shelters was 46.0°C, compared to only 17.0°C under the wooden boards, with shelters reaching a daily average maximum temperature of 35.1°C (SD = 10.0°C) (Figure 1). Shelters reached an average maximum temperature of ≥ 25°C for at least 1 h on 33 of 40 days (ca. 82.5%) and spent an average of 4.2 h above 25°C per day across the monitoring period (Figure S3). The maximum daily average temperature within the shelters exceeded the maximum daily ambient temperature by an average of 17.5°C. Daily global solar exposure averaged 9.3 MJ/m^2^ across the monitoring period (SD = 2.8 MJ/m^2^), reaching a maximum of 12.1 MJ/m^2^ across the monitoring period and exceeding 10 MJ/m^2^ on 21 of 40 days (ca. 52.5%) (Figure 1). Rainfall was recorded on 18 of 40 days (ca. 45.0%) with the daily average rainfall being 1.4 mm (SD = 2.7 mm). The highest rainfall recorded in 1 day was 11 mm.

Daily maximum shelter temperatures were strongly influenced by both solar exposure and ambient temperature (GLS: *β*_solar_ = 3.23 ± 0.25, *β*_ambient_ = 1.24 ± 0.25, both *p* < 0.001; Φ = 0.42), indicating that shelters amplified daily weather variation by more than 1°C for each 1°C increase in ambient conditions and over 3°C per additional MJ/m^2^ of solar input. In contrast, wood temperatures varied only weakly with weather (*β*_solar_ = 0.14 ± 0.03, *β*_ambient_ = 0.13 ± 0.04), reflecting greater thermal stability (Figure S5). The number of hours per day that shelters exceeded 25°C also increased significantly with both ambient temperature and solar exposure (*p* < 0.01).

### 3.2 Werribee Site

Ambient temperatures were much cooler than in Sydney, with the average maximum ambient temperature across both periods being 14.3°C (SD = 2.0°C) and the highest daily maximum ambient temperature reaching only 19.1°C. The average daily minimum temperature was 6.2°C (SD = 3.0°C), with the lowest daily minimum recorded reaching −1.1°C. Temperatures beneath wooden boards never rose higher than 17.7°C (Figure 1), with an average daily maximum of only 8.8°C (SD = 3.4°C). The maximum daily average temperature recorded within the shelters was 29.3°C (SD = 4.2°C). Shelters reached an average maximum temperature of > 25°C for at least an hour on only 8 of 61 days (ca. 13.1%), with an average of only 0.32 h per day spent above 25°C (Figure S3). The maximum daily average temperature within the shelters exceeded the maximum daily ambient temperature by an average of 6.3°C. Daily global solar exposure averaged 8.4 MJ/m^2^ (SD = 2.2 MJ/m^2^), reaching a maximum of 13.1 MJ/m^2^ and exceeding 10 MJ/m^2^ on 17 of 61 days (ca. 27.9%) (Figure 1). Rainfall was recorded on 30 of 61 days (ca. 49.2%), averaging 1.1 mm per day (SD = 2.0 mm) with the maximum rainfall recorded in 1 day reaching 10 mm.

Shelter temperatures were primarily driven by solar radiation (*β*_solar_ = 1.30 ± 0.21, *p* < 0.001), whereas ambient temperature had little effect (*p* = 0.74). Wood temperatures showed the opposite trend, varying mainly with ambient temperature (*β*_ambient_ = 0.46 ± 0.08, *p* < 0.001) and not with solar exposure (*p* = 0.64) (Figure S5). Neither variable significantly predicted the limited time that shelters exceeded 25°C (*p* > 0.4).

## 4 Discussion

Our results indicate that on most days throughout the cooler months of the year a hotspot shelter at the Werribee site or in equivalent climates will create a thermal regime that may worsen disease outcomes by increasing the rate of growth of *Bd* without reaching the upper critical temperature at which *Bd* growth decreases in vitro (~25°C). Within each site, daily maximum temperatures inside shelters were strongly related to concurrent weather conditions, particularly solar exposure, whereas temperatures beneath wooden boards showed much weaker responses (Figure S5).

**FIGURE 2:**
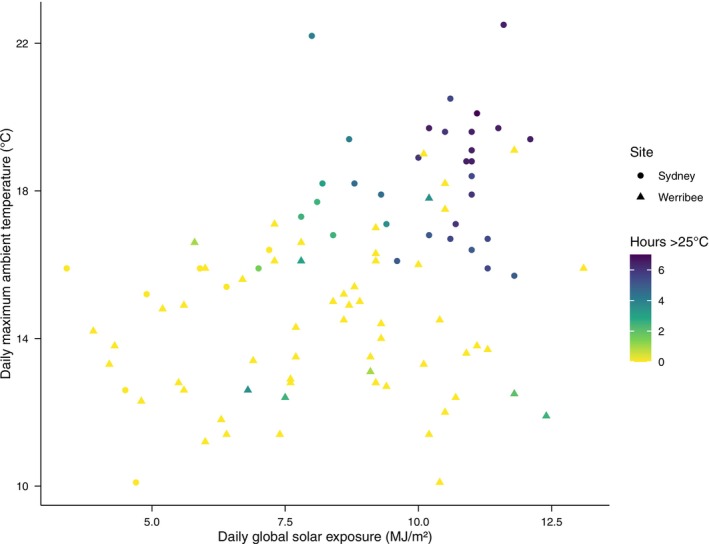
Daily solar exposure and maximum ambient temperature at each site. The Sydney site is shown with circular points and the Werribee site is shown with triangular points. Colour indicates the number of hours spent above 25°C at the corresponding conditions, with darker colours indicating a greater number of hours.

As with previous studies (Waddle et al. 2024), the crevices inside brick piles within a greenhouse were warmer than under a wooden cover‐object. The critical issues for mitigating *Bd*, however, are how much warmer, and for how long per day, and under what range of ambient conditions? Our hotspot shelters in Sydney maintained high temperatures for hours per day even in cool weather, exceeding 25°C for at least 1 h on ca. 82.5% of days (Figure 2, Figure S4), conditions that improved disease outcomes in the study by Waddle et al. (2024). Shelter temperature increased by approximately 1°C for every 1°C rise in ambient temperature and by more than 3°C for each additional MJ/m^2^ of daily solar exposure in Sydney, while wooden boards responded an order of magnitude more weakly (Figure S5). These results demonstrate that the shelters amplified, rather than merely tracked, local weather variation (Figure 1). Hotspots deployed in Werribee did not perform as consistently, with shelters averaging only 0.31 h per day above 25°C and exceeding 25°C for at least 1 h on only ca. 13.1% of days (Figures 1 and 2). We note that 25°C is used here as a conservative indicator of conditions likely to suppress *Bd*. Time spent near 20°C–25°C may still favour pathogen growth, and short periods above 25°C may not fully offset those effects. Thus, this threshold should be interpreted as a comparative index of potential *Bd* suppression rather than a strict biological cutoff. Our characterisation of the temperature profile of shelters in Sydney under conditions previously shown to mitigate chytridiomycosis can inform deployment of this management approach.

While these results describe the thermal environment available within shelters, they do not directly represent the body temperature (Tb) that frogs would experience. Frogs can behaviourally thermoregulate within shelters, and factors, such as aggregation, body mass, posture and skin moisture can produce small, but biologically meaningful, deviations in Tb from ambient conditions. Consequently, the cumulative hours above or below thresholds inferred from dataloggers should be interpreted as approximations of potential rather than realised exposure. Future studies incorporating operative‐temperature models (e.g., agar or copper models) or direct Tb measurements could quantify this relationship and assess fine‐scale heterogeneity within shelters.

The value of hotspot creation for conservation depends on ambient conditions; there is no point deploying hotspot shelters in areas that are too cool or too warm, or do not receive adequate solar exposure. In our study, the benefits of artificial refuges were greatest (i.e., temperatures to > 25°C for at least 1 h) on days with ambient maxima above 15.5°C and when daily solar radiation exceeded 7 MJ/m^2^. These conditions were met on 34 of 40 days at the Sydney site (ca. 85.0%), with shelters maintaining temperatures > 25°C for at least 1 h on 33 of those 34 days (ca. 97.1%). However, whilst these conditions were met at the Werribee site on 14 of 61 days during our study (ca. 23.0%), the shelters only maintained temperatures above 25°C for at least 1 h on two of those 14 days (ca. 14.3%). The remaining 6 days on which shelters at the Werribee site reached > 25°C for at least 1 h had either lower solar radiation (5.8–6.8 MJ/m^2^) or lower maximum temperatures (11.9°C–13.1°C) (Figure 2).

Our study focused on mid‐winter conditions, when *Bd* prevalence typically peaks. Shelter performance outside this period may differ substantially as seasonal changes in solar exposure and temperature alter heating potential. Interannual variability in these climatic factors could likewise influence outcomes. Assessing the performance of hotspots across seasons, especially at sites with year‐round *Bd* pressure, would further clarify the temporal limits of this management approach.

At the Werribee site, even on days when solar exposure and ambient temperature were comparable to that of the Sydney site, shelter warming was less pronounced, consistent with our model showing weaker slopes for both predictors (Figure 2, Figure S5). This difference may be due to overall lower ambient temperatures at Werribee resulting in greater heat loss to the environment. Werribee is also a lower‐humidity environment than Sydney (Beck et al. 2018), which may have contributed to more rapid heat loss from the shelters in Werribee. Although data on wind speed were not collected, the Werribee site appeared to be substantially windier than the Sydney site (e.g., one greenhouse was damaged beyond repair due to high winds towards the end of the monitoring period). High winds would further increase heat loss within the Werribee shelters. Thus, information on solar radiation and ambient temperature alone may not be sufficient to accurately predict the thermal performance of shelters. The relationship between local climatic conditions, the thermal performance of our shelters, and their suitability for a certain species requires further investigation to elucidate what combination of factors will best predict thermal performance and thus enable accurate predictions of where shelters risk becoming ecological traps.

The usefulness of hotspot shelters will differ among species as well as among sites because of interspecific differences in inclination to use hot refuges, as well as the costs and benefits associated with such use at different sites. For example, the temperatures achieved in our Sydney trials are above voluntary thermal maxima, and even above critical thermal maxima, for many cool‐climate amphibians and some reptiles (Duarte et al. 2012; Urban et al. 2014). Host responses to disease also vary among species and environmental conditions. Whilst the thermal performance of *Bd* in culture is relatively consistent, with growth being suppressed at and above ~25°C, and death occurring between 29°C and 30°C (Longcore et al. 1999; Stevenson et al. 2013; Voyles et al. 2017; Turner et al. 2021; Kásler et al. 2022), disease intensity and impact are not reliably predicted by environmental conditions such as air temperature in situ (Sonn et al. 2017; Turner et al. 2021). Indeed, the duration of elevated temperature required to reduce or eliminate *Bd* infections in vivo remains poorly defined and varies widely. Although 29°C–30°C has consistently been reported as the critical thermal maximum of *Bd* in vitro, Holmes et al. (2024) reported reoccurrence of detectable infections in four of 13 frogs when returned to temperatures between 21°C and 29°C, despite having been held between 29°C and 34°C for 4 weeks. Similarly, Chatfield and Richards‐Zawacki (2011) reported successful clearance in only 26 of 27 individuals held at 30°C for 10 days. Together, these results suggest that the consistent upper thermal limit of *Bd* reported across in vitro studies may not directly translate to in vivo outcomes (see Table 1; Holmes et al. (2024) for a detailed summary of in vivo outcomes at various elevated temperatures).

Complex interactions between factors such as environmental conditions and host immune function (Rollins‐Smith 2020), host skin microbiome (Daskin et al. 2014), host thermoregulatory behaviour (Stevenson et al. 2020) and pathogen growth must be considered holistically. For example, it may be that access to varied temperatures is more effective in reducing *Bd* infection loads than exposure to one constant high temperature in vivo. Waddle et al. (2024) found that infection intensity reduced more rapidly in *L. aurea* kept in thermal gradients where they were able to freely thermoregulate, than in those kept at constant high or low temperatures.

This complexity makes it difficult to predict disease outcomes across species based solely on ambient temperatures. Whilst our shelters in Werribee did not maintain temperatures high enough to reduce infections based on in vitro thermal performance of *Bd*, they did still elevate temperatures to substantially above both the daily maximum ambient temperature and the daily maximum temperature achieved beneath wooden boards (Figure 1, Figure S5). For host species in cool regions, these increases may be adequate to reduce disease burden if access to a wider range of temperatures allows for more effective thermoregulatory behaviour, and in turn enables improved host immune function. Moreover, full clearance of infection may not be necessary if elevated temperatures within shelters enable frogs to suppress infections below levels that result in disease and remain asymptomatic through critical winter periods (Campbell et al. 2019; Holmes et al. 2024; Waddle et al. 2024).

Questions remain regarding how artificial shelters could change frog behaviours and aggregation, and hence impact disease transmission. Where thermal preferences differ among sexes, sizes or species (Yang et al. 2019), added shelters could reduce spatial overlap of such subgroups. For example, sympatric amphibian species may co‐occupy refugia when conditions are uniform but segregate when heterogeneity increases. This segregation might reduce interspecific pathogen transfer, a major risk for chytrid‐vulnerable species infected by proximity to non‐vulnerable carriers (Burns et al. 2021). A cold‐adapted vulnerable species could benefit if hotspots draw either that species or carriers into separate refugia, but, conversely, warmer retreats might aggregate species that usually remain apart, heightening cross‐infection risk.

Some of the disadvantages of hotspots in cool‐weather sites could be reduced through engineering changes to increase temperature more substantially. Thus, future work could explore not only the thermal effects of brick piles, but also other shelters with more active heating. Alternative sources of heating such as solar panels (Hettyey et al. 2019) and reticulation of water through decomposing compost (Nwanze and Clark 2019) might work in some situations. Shapes that are less susceptible to uplift from wind (e.g., domes) also may reduce heat loss in windy environments, especially if these structures incorporate insulative materials. Regardless, it is first necessary to determine what thermal regimes are required for target species to reduce their disease burdens, and thus what range of temperature increases are required within shelters, on a case‐by‐case basis. It is not reasonable to assume that the in vitro thermal performance of *Bd* also applies in vivo, uniformly, across all species and in all environments.

There will be no ‘one size fits all’ approach to creating and deploying thermal refugia. Benefits of Waddle et al.'s (2024) hotspot system likely will be greatest for thermophilic species that inhabit relatively open (and thus, sun‐exposed) habitats in climates that are neither severely hot nor severely cold, and in which the existing array of potential refuge sites incorporates little thermal variation. But the general approach, of amplifying the range of abiotic conditions available in refuges, should be applicable to a diversity of threatened taxa. Given the central role of ambient thermal regimes in ectotherm biology, manipulating thermoregulatory opportunities may offer a simple and powerful way to enhance organismal viability. Although such a tactic is labour‐intensive, it is well suited to the growing involvement of the general community in wildlife conservation—with the caveat that we must adjust approaches to local conditions. By testing performance of hotspot shelters at a colder site, we suggest that such shelters may have negative rather than positive impacts on frog health if deployed in inappropriate environments.

## Author Contributions

**Madeleine L. Holmes:** conceptualization (supporting), data curation (lead), formal analysis (lead), visualization (lead), writing – original draft (equal), writing – review and editing (equal). **Shannon W. Kaiser:** data curation (supporting), investigation (equal), writing – review and editing (equal). **Sneha S. Basu:** conceptualization (equal), investigation (equal), writing – review and editing (equal). **Lee Berger:** conceptualization (equal), investigation (supporting), methodology (equal), supervision (equal), writing – review and editing (equal). **Lee F. Skerratt:** conceptualization (equal), investigation (supporting), supervision (equal), writing – review and editing (equal). **Richard Shine:** conceptualization (equal), data curation (supporting), formal analysis (supporting), investigation (supporting), methodology (equal), supervision (equal), writing – original draft (equal), writing – review and editing (equal). **Anthony W. Waddle:** conceptualization (equal), investigation (equal), methodology (equal), project administration (equal), supervision (equal), writing – review and editing (supporting).

## Conflicts of Interest

The authors declare no conflicts of interest.

## Supporting information


**Figure S1:** ece372590‐sup‐0001‐Supinfo.docx.
**Figure S2:** ece372590‐sup‐0001‐Supinfo.docx.
**Figure S3:** ece372590‐sup‐0001‐Supinfo.docx.
**Figure S4:** ece372590‐sup‐0001‐Supinfo.docx.
**Figure S5:** ece372590‐sup‐0001‐Supinfo.docx.
**Table S1:** ece372590‐sup‐0001‐Supinfo.docx.
**Table S2:** ece372590‐sup‐0001‐Supinfo.docx.

## Data Availability

The data supporting this research are openly available in Dryad at https://doi.org/10.5061/dryad.0zpc867b6. Ambient weather data were obtained from the Australian Bureau of Meteorology (BOM) Climate Data Online portal, which is publicly accessible at: http://www.bom.gov.au/climate/data.
