# Supplementary material for: Disease Refuge or Ecological Trap: Location‐Specific Performance of Amphibian Hotspot Shelters
Source: Ecol Evol. 2026 Mar 30;16(4):e72590. doi: 10.1002/ece3.72590 (PMC13107285; doi:10.1002/ece3.72590)
Supplement: Supplementary file 1 — Figure S1: ece372590‐sup‐0001‐Supinfo.docx. Figure S2: ece372590‐sup‐0001‐Supinfo.docx. Figure S3: ece372590‐sup‐0001‐Supinfo.docx. Figure S4: ece372590‐sup‐0001‐Supinfo.docx. Figure S5: ece372590‐sup‐0001‐Supinfo.docx. Table S1: ece372590‐sup‐0001‐Supinfo.docx. Table S2: ece372590‐sup‐0001‐Supinfo.docx. [file ECE3-16-e72590-s001.docx]

**Figure S1** Map indicating the location of the two sites within Australia. The circular point indicates the sub-tropical Sydney site, and the triangular point indicates the temperate Werribee site.

**Figure S2** Photographs of the experimental refuges, showing (a) two shelters at the Werribee site, (b) the configuration of the bricks within shelters with stars indicating location of temperature loggers, and frogs (*Litoria aurea*) sheltering within bricks at the Sydney site (c).


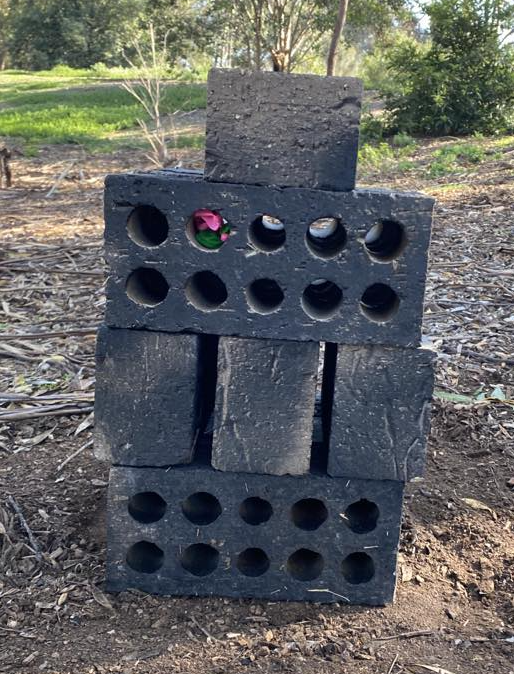

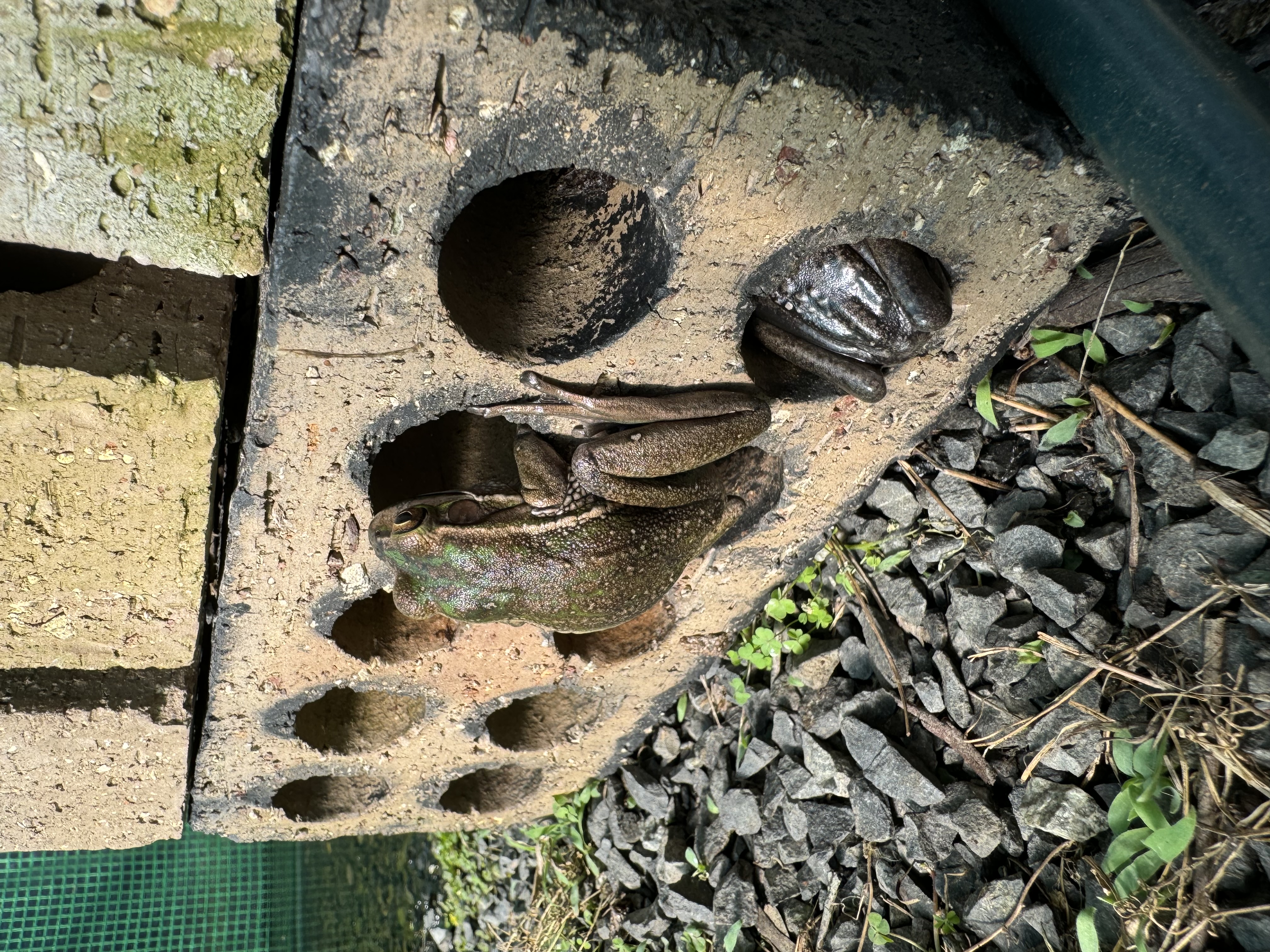

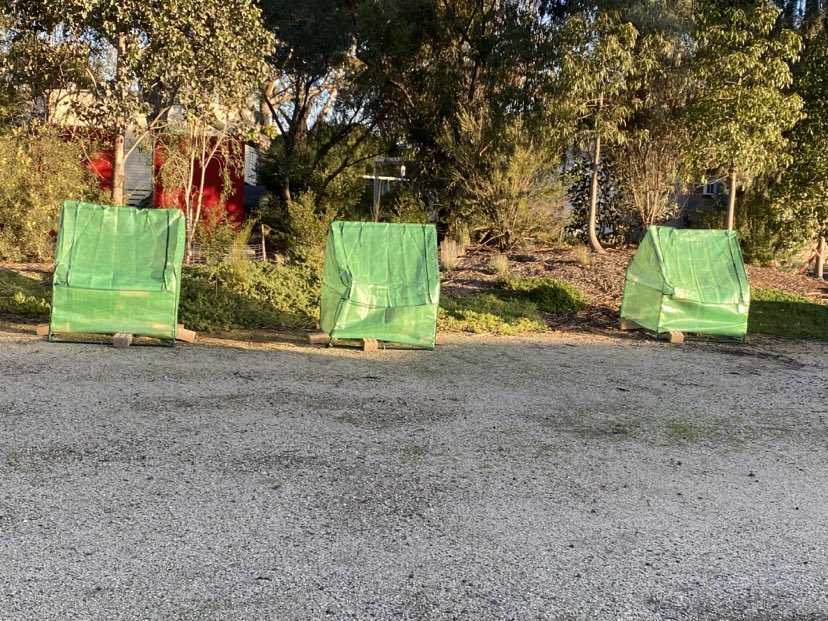


**a**

**b**

**c**

**Figure S3** Average hours per day that dataloggers spent above 20 degrees (a) and 25 degrees (b) for three maximum daily ambient temperature ranges. Solid lines represent values from within shelters, dashed lines indicate values from under wooden boards. Error bars show calculated standard error. Note that daily maximum ambient temperature did not exceed 20°C at the Werribee site, and loggers beneath wood never exceeded 25°C at either site.

**
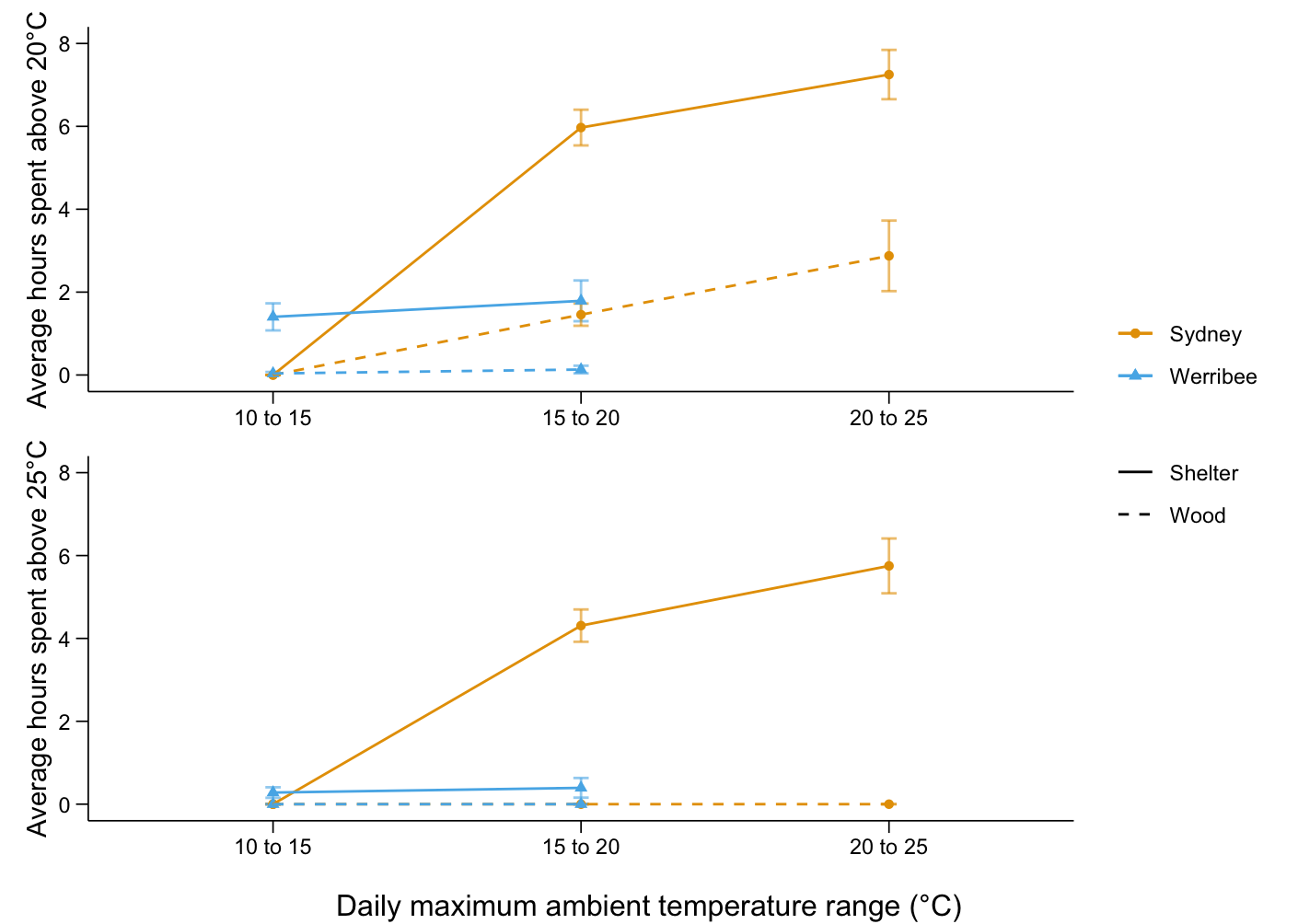
**

**a**

**b**

**Figure S4** Cumulative distribution of percentage of days shelters spent X hours above 25°C for each site. Orange solid line is the Sydney site, dashed blue line is the Werribee site.


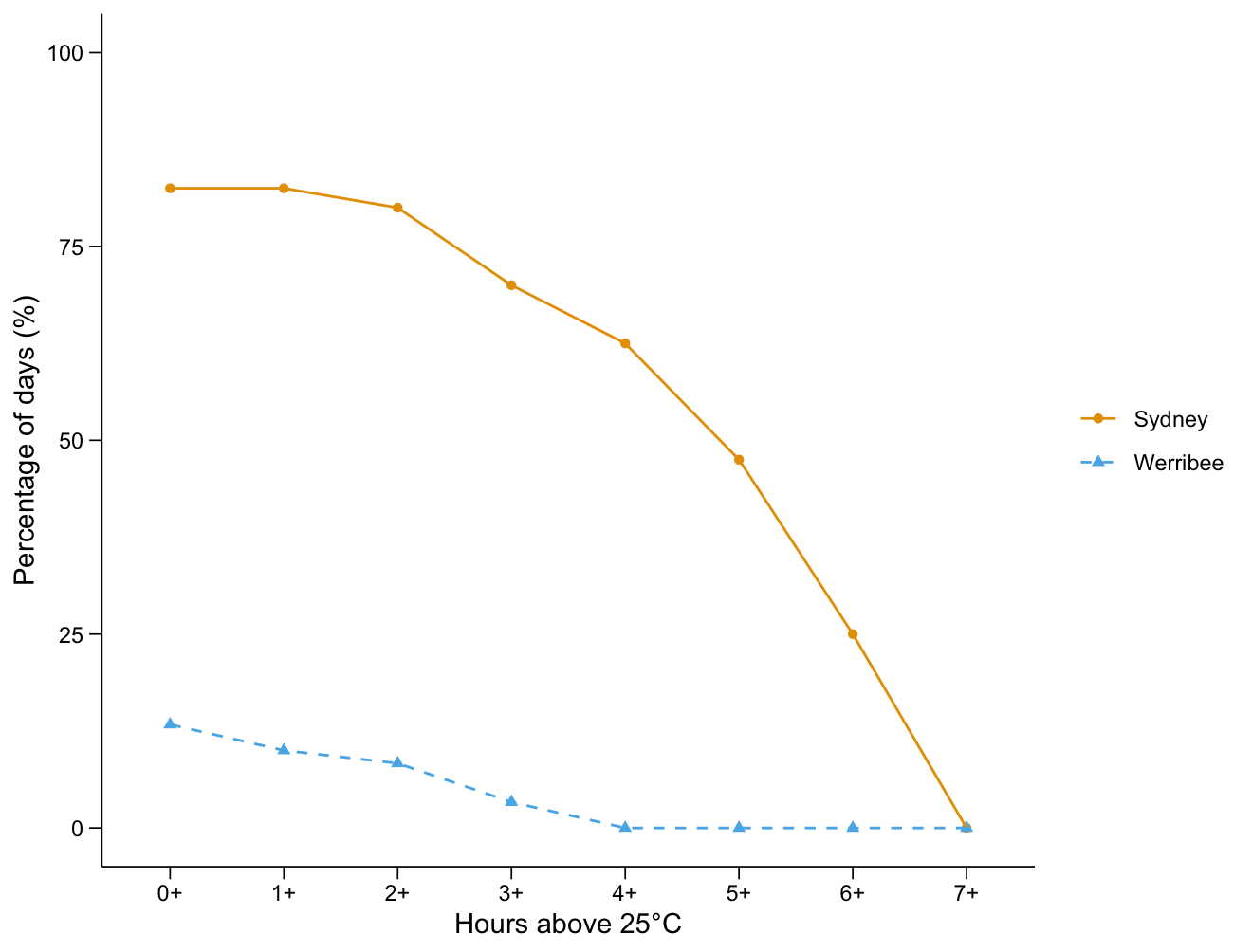


**Figure S5** Shelter heating advantage over amphibian retreat analogue (wooden board on ground).

| **Station name** | **Station number** | **Elevation (m)** | **Coordinates** |
| --- | --- | --- | --- |
| Marsfield (Macquarie University No:2) | 66193 | 51 | 33.77° S 151.12° E |
| Macquarie Park (Willandra Village) | 66156 | 65 | 33.78° S 151.11° E |
| Sydney Olympic Park AWS (Archery Centre) | 66212 | 4 | 33.83° S 151.07° E |
| Werribee (Racecourse) | 87150 | 20 | 37.90° S 144.64° E |
| Point Cook RAAF | 87185 | 3 | 37.93° S 144.76° E |
| Laverton RAAF | 87031 | 20 | 37.86° S 144.76° S |

**Table S1** Australian Bureau of Meteorology weather stations used for climate data

| **Start date** | **End date** | **Station number** | **Solar exposure** | **Rainfall** | **Max temp** | **Min temp** |
| --- | --- | --- | --- | --- | --- | --- |
| 10/6/2021 | 19/7/2021 | 66193 | ***** |  |  |  |
| 10/6/2021 | 19/7/2021 | 66156 |  | ***** |  |  |
| 10/6/2021 | 19/7/2021 | 66212 |  |  | ***** | ***** |
| 18/6/2022 | 20/8/2022 | 87150 | ***** |  |  |  |
| 18/6/2022 | 5/7/2022 | 87185 |  | ***** | ***** | ***** |
| 10/7/2022 | 13/7/2022 | 87031 |  | ***** |  |  |
| 10/7/2022 | 12/7/2022 | 87031 |  |  | ***** | ***** |
| 14/7/2022 | 20/8/2022 | 87185 |  | ***** |  |  |
| 13/7/2022 | 20/8/2022 | 87185 |  |  | ***** | ***** |

**Table S2** Data type sourced from each weather station by date
